# Integrating Melt Electrowriting and Fused Deposition Modeling to Fabricate Hybrid Scaffolds Supportive of Accelerated Bone Regeneration

**DOI:** 10.1002/adhm.202302057

**Published:** 2023-11-16

**Authors:** Kian F. Eichholz, Pierluca Pitacco, Ross Burdis, Farhad Chariyev‐Prinz, Xavier Barceló, Brooke Tornifoglio, Ryan Paetzold, Orquidea Garcia, Daniel J Kelly

**Affiliations:** ^1^ Trinity Centre for Biomedical Engineering Trinity Biomedical Sciences Institute Trinity College Dublin 152‐160 Pearse Street Dublin D02 R590 Ireland; ^2^ Department of Mechanical, Manufacturing and Biomedical Engineering School of Engineering Trinity College Dublin Dublin D02 VH29 Ireland; ^3^ Advanced Materials and Bioengineering Research Centre (AMBER) Royal College of Surgeons in Ireland and Trinity College Dublin Dublin D02 CP49 Ireland; ^4^ School of Mechanical and Materials Engineering University College Dublin Dublin D04 E4X0 Ireland; ^5^ Johnson & Johnson 3D Printing Innovation and Customer Solutions Johnson & Johnson Services, Inc. Irvine CA 92618 USA; ^6^ Department of Anatomy and Regenerative ME Royal College of Surgeons in Ireland Dublin D02 YN77 Ireland

**Keywords:** 3D printing, angiogenesis, bone repair, fused deposition modeling, melt electrowriting, scaffold architecture

## Abstract

Emerging additive manufacturing (AM) strategies can enable the engineering of hierarchal scaffold structures for guiding tissue regeneration. Here, the advantages of two AM approaches, melt electrowriting (MEW) and fused deposition modelling (FDM), are leveraged and integrated to fabricate hybrid scaffolds for large bone defect healing. MEW is used to fabricate a microfibrous core to guide bone healing, while FDM is used to fabricate a stiff outer shell for mechanical support, with constructs being coated with pro‐osteogenic calcium phosphate (CaP) nano‐needles. Compared to MEW scaffolds alone, hybrid scaffolds prevent soft tissue collapse into the defect region and support increased vascularization and higher levels of new bone formation 12 weeks post‐implantation. In an additional group, hybrid scaffolds are also functionalized with BMP2 via binding to the CaP coating, which further accelerates healing and facilitates the complete bridging of defects after 12 weeks. Histological analyses demonstrate that such scaffolds support the formation of well‐defined annular bone, with an open medullary cavity, smooth periosteal surface, and no evidence of abnormal ectopic bone formation. These results demonstrate the potential of integrating different AM approaches for the development of regenerative biomaterials, and in particular, demonstrate the enhanced bone healing outcomes possible with hybrid MEW‐FDM constructs.

## Graphical abstract

This work outlines the development of a hybrid scaffold consisting of a micro‐fibrous core made using melt electrowriting (MEW) and an outer shell made using fused deposition modeling (FDM). This study demonstrates the potential of integrating different additive manufacturing approaches for the development of regenerative biomaterials, and in particular, demonstrates the enhanced bone healing outcomes possible with hybrid MEW‐FDM constructs.

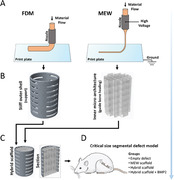

## 1 Introduction

Additive manufacturing (AM) techniques are increasingly being used for the fabrication of scaffolds for tissue and organ regeneration. This is driven by the relative ease by which these techniques can be used to fabricate constructs with exterior geometries to fit a particular defect, as well as internal architectures designed to elicit a specific mechanical or biological response. AM devices to aid bone regeneration have progressed to the stage of FDA approval, including the DePuy Synthes TruMatch Graft Cage which is composed of polycaprolactone (PCL) and hydroxyapatite (HA).^[^ ^1^ ^]^ PCL is commonly used in the AM of regenerative scaffolds, due in part to its rheological and viscoelastic properties which contribute to its ease of processing for AM, its biodegradable nature, and its relatively low cost.^[^ ^2^, ^3^ ^]^ Polymers like PCL can be processed into scaffolds using diverse AM techniques. Each approach has its relative strengths and drawbacks, and may be limited for example in terms of the mechanical strength of the resulting construct, the complexity of geometries which can be fabricated, or the minimum feature size which can be achieved. This in turn limits the design space by which complex hierarchical architectures can be fabricated to guide bone regeneration.^[^ ^4^ ^]^ Combining multiple AM techniques would greatly expand this design space, potentially enabling the fabrication of more complex scaffolds capable of accelerating tissue regeneration.^[^ ^5^ ^]^

One of the most commonly used AM approaches for fabricating tissue engineering (TE) scaffolds is fused deposition modelling (FDM), where molten material is deposited in layer‐by‐layer fashion based on user defined print paths. This provides precise control over scaffold architecture, which from a bone TE perspective, dictates mechanical properties, nutrient transport, and cellular fate for different scaffolding materials.^[^ ^6^, ^7^ ^]^ Models can also be generated from computer tomography (CT) or magnetic resonance imaging (MRI) scans to reconstruct and print personalized implants.^[^ ^8^, ^9^, ^10^ ^]^ This concept was demonstrated in a recent study which aimed to guide bone healing in several complex long bone defects in humans.^[^ ^11^ ^]^ Here, CT scans were conducted to produce 3D models of the defects, and the inner structure was based on a repeating triangular pore architecture. Scaffolds were then printed with a PCL‐tricalcium phosphate blend, filled with autologous bone graft material and then implanted, with promising outcomes observed in terms of bone ingrowth and patient weight bearing. Another emerging extrusion‐based AM approach is melt electrowriting (MEW).^[^ ^12^, ^13^ ^]^ With this technology, material is also laid down in a layer by layer fashion, however, much lower fiber diameters (in the micron to sub‐micron diameter range) can be achieved.^[^ ^14^, ^15^ ^]^ This in turn allows the fabrication of highly porous structures with up to 98% porosity,^[^ ^16^ ^]^ in addition to high specific surface areas,^[^ ^17^ ^]^ which has multiple benefits including enhanced cell attachment,^[^ ^18^, ^19^ ^]^ increased protein adsorption sites,^[^ ^20^ ^]^ and enhanced oxygen delivery to cells.^[^ ^21^ ^]^ This greatly opens up the design space for producing TE scaffolds, and allows the fabrication of intricate micro‐fibrous geometries to guide cell behavior and elicit a desired response for bone regeneration.^[^ ^22^, ^23^, ^24^ ^]^ Indeed, using MEW technology, architectural features much closer to those seen in native bone are achievable,^[^ ^15^, ^25^ ^]^ namely, its hierarchical fibrous microstructure including collagen fibrils with approximate diameter of 500 nm, collagen fibers in the range of 1–10 µm in diameter, and the porous open network of trabecular bone with pore sizes in the order of 0.5–1 mm.^[^ ^4^ ^]^ However, MEW has several limitations, with one being the typically long time it takes to print large volume scaffolds, and another being the typically low stiffness and spongy characteristic of scaffolds, which reduces their utility for load bearing applications.

In addition to the physical architecture of a TE scaffold, its composition is also critically important for effective bone repair.^[^ ^26^, ^27^ ^]^ The mineral structure of bone is fundamentally composed of nanoscale, needle‐shaped CaP units, which in turn form platelets spread through a fibrous collagen matrix.^[^ ^28^ ^]^ Applying a nanoscale, needle‐shaped mineral structure to TE scaffolds has been shown to enhance mesenchymal stem cell (MSC) osteogenesis in vitro^[^ ^25^ ^]^ and supports bone formation in vivo.^[^ ^29^ ^]^ Additionally, nanostructured minerals can stabilize and control the release of proteins and other bioactive factors, resulting in enhanced healing.^[^ ^30^ ^]^ Growth factors such as bone morphogenic proteins (BMPs) are commonly used to great effect for bone regeneration.^[^ ^31^, ^32^, ^33^, ^34^, ^35^ ^]^ For example, BMP2 has been combined with autologous bone graft material inside a 3D printed scaffold implanted in a long bone defect in a human.^[^ ^11^ ^]^ However, use of BMP2 is associated with various adverse effects such as inflammation and ectopic bone formation.^[^ ^33^ ^]^ Excessive BMP2 concentrations and leakage outside of the implant site may be the leading causes of such adverse events,^[^ ^33^ ^]^ motivating the development of delivery systems for controlled growth factor release.^[^ ^36^, ^37^ ^]^ Factors such as BMP2 could be used to functionalize 3D printed scaffolds via binding to a mineral coating as has previously been demonstrated,^[^ ^25^ ^]^ allowing sustained release and enhancing the ability of such scaffolds to safely support bone regeneration.

**Figure 1:**
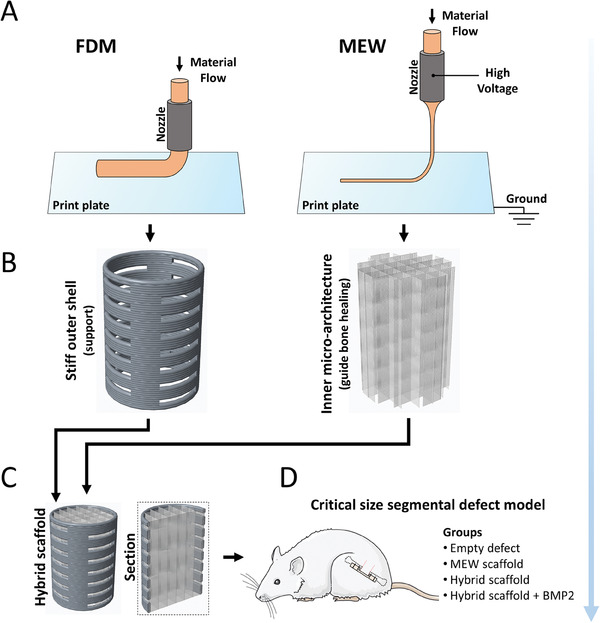
Study outline. A) Fused deposition modelling (FDM) and melt electrowriting (MEW) 3D printing methods were used for the fabrication of bone tissue engineering scaffolds. B) FDM was used to fabricate a stiff outer structure with side porosity while MEW was used to fabricate the inner micro‐fibrous architecture. C) Scaffolds were assembled after the FDM and MEW printing processes. D) Bone healing was assessed using a critically sized segmental femoral defect model in rats.

We have previously observed that MEW can be used to create more osteogenic scaffolds with enhanced bone healing compared to those with comparable surface area fabricated by FDM.^[^ ^17^ ^]^ We have also observed that a potential benefit of FDM scaffolds was their ability to produce more rigid structures, which during large bone defect healing, acted to limit surrounding muscle/soft tissue collapse into the defect region and increase the volume available for new bone healing. Recognizing the relative benefits of both AM approaches, here we sought to develop a hybrid scaffold for large bone defect healing. This hybrid scaffold consisted of an outer shell fabricated using FDM, which functioned to mechanically support the healing defect and limit soft tissue collapse, along with an inner core produced by MEW, which functioned to guide the bone regeneration process (**Figure** **1**). Furthermore, we sought to apply an osteogenic calcium phosphate (CaP) coating to these hybrid scaffolds and functionalize them with BMP2 (via binding to the CaP) to accelerate bone regeneration. Scaffolds were implanted into critically sized segmental rat femoral defects to assess their ability to guide bone healing in vivo.

## 2 Experimental Section

### 2.1 Scaffold Fabrication

#### 2.1.1 Study Outline

Hybrid scaffolds were fabricated by assembling a MEW core within an FDM shell and coating with CaP, with one group being additionally functionalized with BMP2. Scaffolds were implanted in critically sized segmental rat femoral defects to assess their ability to guide large bone defect healing in vivo. The aim of this study was to assess the potential of hybrid scaffolds to regenerate bone compared to MEW scaffolds alone, and additionally, to assess the performance of a hybrid scaffold functionalized with BMP2 with the aim of development toward clinical applications.

#### 2.1.2 Scaffold Design

Scaffold concepts were initially designed using Creo Parametric 3.0 computer aided design (CAD) software. The hybrid scaffold concept is a two‐part manufacturing process, where the core of the scaffold is fabricated using MEW, and the shell of the scaffold is manufactured using FDM. Both of these components were subsequently assembled, with MEW fibers laterally protruding through the side pores of FDM shells, locking the entire assembly securely in place. Scaffold groups consisted of MEW scaffolds alone, and hybrid MEW‐FDM scaffolds. A third scaffold group was also created by functionalizing hybrid MEW‐FDM scaffolds with BMP2.

#### 2.1.3 Melt Electrowriting 3D Printing

MEW scaffolds were printed on a custom MEW 3D printer built in house as previously described.^[^ ^15^ ^]^ Briefly, PCL (CAPA 6500D) was loaded in a syringe barrel and pre‐melted at 90 °C overnight in an oven to remove air bubbles. A 22G nozzle of length 6.35 mm (Nordson EFD, 7018260) was attached to the barrel before being mounted in the print head. Temperatures of 85 and 80 °C were used for the nozzle and barrel heaters respectively. An air pressure of 0.08 MPa was used for extrusion and a collector speed of 6 mm s^−1^ was used for printing. Scaffolds were printed with an initial nozzle height of 8 mm and voltage of 7 kV, which were incrementally increased each layer to result in a final nozzle height of 13 mm and voltage of 9.84 kV. Scaffolds of height 4.75 mm and diameter 3.75 mm were cut using a Piranha CO_2_ laser cutter (BCL‐0503MUF) at a speed of 90 mm s^−1^, power of 20%, and low air assist control. Five passes of cuts were taken to cut completely through MEW scaffolds.

#### 2.1.4 Fused Deposition Modeling 3D Printing

FDM shells were fabricated using a RegenHU 3DDiscovery printer. PCL (CAPA 6500D) was printed using a 30G nozzle with a temperature of 82 °C at the head and pressure of 0.25 MPa. Scaffolds were designed to have an inner diameter of 3.7 mm when printed, and outer diameter of 4.3 mm, with the print path showing how fibers were placed shown in Figure S1, Supporting Information. FDM shells were printed to a height of 4.75 mm. MEW and FDM scaffolds were then manually assembled by placing MEW scaffolds within FDM shells. MEW scaffolds were kept securely in place due to being slightly larger than the inner diameter of the FDM shell, and due to MEW fibers protruding slightly through the side pores of the FDM shells, latching the MEW scaffold in place.

#### 2.1.5 Coating with Nano‐Needle Hydroxyapatite (nnHA)

MEW and hybrid scaffolds were coated in hydroxyapatite with a needle shaped nanostructure (nnHA) as previously described,^[^ ^25^ ^]^ with a total of 12 coatings. Briefly, scaffolds were placed in 70% ethanol at room temperature for 15 min and a vacuum was applied to remove air bubbles. Scaffolds were then immersed in 2 m NaOH for 5 min under vacuum, and then further incubated without vacuum at 37 °C for 45 min. Scaffolds were then rinsed five times in MilliQ water. Scaffolds were immersed in a 0.05 m calcium chloride dihydrate solution, before an equal volume of 0.03 m sodium phosphate tribasic dodecahydrate solution was added dropwise. A vacuum was applied for 5 min and scaffolds were then incubated for 30 min at 37 °C. The coating procedure from the stage of immersion in calcium chloride dihydrate was then repeated 11 more times, minus the vacuum step, resulting in a total of 12 mineral coatings. Scaffolds were then allowed to dry overnight.

### 2.2 Scanning Electron Microscopy

Scaffolds were mounted on pin stubs with adhesive carbon tabs, and then coated with gold/palladium for 60 s at a current of 40 mA using a Cressington 208HR sputter coater. Imaging was carried out in a Zeiss ULTRA plus SEM with an accelerating voltage of 5 kV, with the SE2 detector being used for lower magnification imaging of scaffolds, and the InLens detector being used for higher magnification imaging of the hydroxyapatite coating.

### 2.3 Mechanical Characterization

Unconfined compression tests were carried out using a single‐column ZwickRoell universal testing machine (ZwickRoell, Germany) with a 200 N load cell. Scaffolds (*n* = 4–5) were compressed at a speed of 0.5 mm min^−1^ between two metal platens up to a maximum of 31.5% strain. Engineering stress was calculated by dividing load values with the total circular cross‐sectional area of scaffolds, and engineering strain was calculated by dividing displacement with the initial scaffold height. The elastic modulus, and in the case of hybrid scaffolds, the modulus in the toe region and linear region, were calculated from the resulting stress–strain curves. Strain was subsequently converted to percent for visualization in stress–strain graphs. Unpaired T‐tests were performed to compare the stiffness of MEW scaffolds to the stiffness of hybrid scaffolds in the toe region, and to compare the stiffness of MEW scaffolds to stiffness of hybrid scaffolds in the subsequent linear region.

### 2.4 BMP2 Loading of Scaffolds

Scaffolds were first sterilized via ultraviolet (UV) light for 1 h on each side. They were further sterilized via immersion in a 70% ethanol solution for 1 h before rinsing three times in sterile deionized water. Scaffolds were then allowed to dry. For the hybrid+BMP2 group, a 40 µL solution containing 10 µg of rhBMP‐2 (PeproTech, UK) was slowly deposited to the MEW core of each hybrid scaffold. This was done in the morning, 1 day before the scheduled surgery day. The solution easily wetted and spread through the scaffold due to the hydrophilic nature of the nnHA coating. Scaffolds were left under sterile conditions for 24 h to allow them to dry before implantation. Upon implantation, scaffolds were predominantly dry with a minimal amount of undried solution present.

### 2.5 BMP2 Release Study and Immunohistochemical Staining

For the BMP2 release study, hybrid+BMP2 scaffolds (*n* = 4) were immersed in 100 µL release medium (consisting of 0.5% bovine serum albumin (BSA) in phosphate buffered saline (PBS)) in a 96‐well plate. Release medium was removed at each time‐point and replenished with fresh medium. Time‐points were as follows: 1 min, 5 min, 60 min, 24 h, 5 days, 7 days, 14 days, 21 days. Release medium samples were stored at −20 °C until the end of the study and all quantified together. BMP2 content was quantified via ELISA (R&D Systems, DY355‐05) as per the manufacturer's instructions. After the release study, immunohistochemical BMP2 staining was conducted on scaffolds to assess if BMP2 was still present after the 21 day study. Scaffolds were washed with PBS (3×5 min) and incubated with blocking buffer (10% goat serum with 1% BSA in PBS) at room temperature for 3.5 h. Scaffolds were then incubated in primary antibody (ab96826, Abcam) with a dilution of 1:200 overnight at 4 °C. Next, the secondary antibody was added (Goat Anti‐Rabbit IgG H&L (Biotin), ab207995, Abcam) with a dilution of 1:250 for 1 h at room temperature, followed by rinsing with blocking buffer (3×5 min) and incubation with ABC reagent (Vectastain PK‐400, Vector Labs) for 45 min. Finally, scaffolds were rinsed with PBS (3×5 min) and developed with DAB peroxidase (Vector Labs).

### 2.6 In Vivo Rat Femoral Segmental Defect Implantation

Implant groups for in vivo studies consisted of empty defect controls (empty), MEW scaffolds (MEW), hybrid scaffolds consisting of an inner MEW portion and outer FDM portion (hybrid), and the former group functionalized with BMP2 (hybrid+BMP2). 34 (*n* = 7 for empty group, *n* = 9 for other groups) 12‐week old male Wistar Han rats were used in this study. Rats were anesthetized in an induction box using a mix of isoflurane and oxygen, with an initial isoflurane flow rate of 5 L min^−1^ to induce anesthesia, followed by a flow rate of 3 L min^−1^ to maintain anesthesia. Animals were then transferred to a 37 °C heating plate covered with a surgical drape, prepared by shaving and cleaning the left hind limb, and administered a pre‐operative analgesia of buprenorphine (0.5 mg kg^−1^). Animals were then transferred to the operating table, and surgical access to the femur was achieved via an anterolateral longitudinal skin incision and separation of the hind limb muscles, the vastus lateralis and biceps femoris. The femoral diaphysis was exposed by circumferential elevation of attached muscles and the periosteum was removed. Before the creation of the defect, a weight‐bearing polyether ether ketone (PEEK) internal fixation plate was secured to the anterolateral femur. Holes were created in the femur with a surgical drill using the holes already present in the PEEK plate as a template. Screws were inserted into holes in the femur immediately after drilling to securely attach the fixation plate. A 5 mm mid‐diaphyseal defect was created using an oscillating surgical saw under constant irrigation with sterile saline solution. Scaffolds were then inserted into the defect following a thorough wash of the surgical site (Figure S2, Supporting Information). Soft tissue was accurately readapted with absorbable suture material. Closure of the skin wound was achieved using sutures. This animal procedure and study was approved by the ethics committee in Trinity College Dublin and the Health Products Regulatory Authority (HPRA) in Ireland (Approval—AE19136/P087). In vivo µCT analysis was performed on rats at 6 and 12 weeks post‐operation (see below). At 12 weeks rats were sacrificed by CO_2_ asphyxiation and the affected femur, with the PEEK plate fixator intact, was excised for further analysis.

### 2.7 In Vivo µCT Imaging and Analysis

A Scanco Medical vivaCT 80 system (Scanco Medical, Bassersdorf, Switzerland) was used to perform micro computed tomography (µCT) scans on rats at 6 and 12 weeks after surgery to assess bone formation in the defect, in addition to bone volume in the healthy leg as a further control at 12 weeks. Anesthesia was induced in an induction box as previously described for the surgical procedure, with an initial isoflurane flowrate of 5 L min^−1^ to induce anesthesia followed by a lower flow rate of 3 L min^−1^ to maintain anesthesia. Rats were then placed inside the µCT scanner, with anesthesia being continuously maintained by an isoflurane‐oxygen mixture at a flow rate of 3 L min^−1^ throughout the scan. A large radiographic scan of lower animal body was performed to locate the rat femur and verify that the femur was appropriately aligned to the scanning field of view to simplify bone volume assessments, with scan slices being imaged transverse to the femur along its long axis. Scans were taken using a voltage of 70 kVp, current of 113 µA and a total of 220 slices per femur. A voxel resolution of 35 µm was used throughout, corresponding to a slice size of the same height (0.0349 mm). A Gaussian filter (sigma = 0.8, support = 1) was used to suppress noise, and a global lower threshold of 282 was applied. 3D evaluation was carried out on the segmented images to determine bone volume (mm^3^), bone density (mg HA ccm^−1^), and to construct 3D models. Bone volume and bone density in the defects were quantified by measuring the total quantity of mineral in the central 5 mm (143 slices) of the defect. Further analyses of bone distribution along this 5 mm length was conducted by quantifying bone volume in a cylindrical core of diameter 2 mm (core region), in a tubular region with inner diameter of 2 mm and outer diameter of 5 mm (annulus region), and in the region outside a cylinder of diameter 5 mm (heterotopic region). Scans of healthy control femurs were used to help define the geometrical values of these regions.

### 2.8 Histological Analysis and Vessel Quantification

Femurs were fixed in 10% neutral buffered formalin for 48 h, and then decalcified using Decalcifying Solution‐Lite (Sigma, D0818) under shaking for 2 weeks. These initial steps were conducted with PEEK plates still attached to maintain integrity of samples during processing. The PEEK plate was then removed, and femurs cut to a length of approximately 15 mm (with the 5 mm defect in the middle). Samples were subsequently dehydrated and embedded in paraffin using an automatic tissue processor (Leica ASP300, Leica). Samples were sectioned with a thickness of 10 µm using a rotary microtome (Leica Microtome RM2235, Leica). Sections were then stained with hematoxylin and eosin (H&E) to assess general tissue morphology, Goldner's Trichrome to assess bone formation and perform quantification on vessel formation and morphology, and Safranin O to assess sGAG content. To quantify vessel formation (vessel number), three different slices each of varying depth within the defect stained with Goldner's Trichrome were analyzed for all rats in each study group. In each rat, this yielded three longitudinal planes throughout the defect at different depths, where vessels were quantified. A 5 mm^2^ region of interest (of dimensions 2.5 × 2.0 mm) was defined in the mid defect region of each slice, with this region containing only newly formed tissue and no pre‐existing bone from the defect ends. Vessels within this area were manually counted and marked using Aperio ImageScope v12.4 software. Results were then normalized to the defined 5 mm^2^ to determine the total number of vessels per square mm. Vessel size was also quantified by measuring the diameter of up to 20 random vessels in 5 rats per group (3 rats for the empty group).

### 2.9 Statistical Analysis

Statistical analyses were performed using GraphPad Prism v9 software. Unless otherwise stated, one‐way or two‐way ANOVA with Tukey's multiple comparisons post‐test were performed where appropriate. Numerical and graphical results are displayed as mean ± standard deviation. * = statistical significance between groups, where the significance level is as follows: * = *p* ≤ 0.05, ** = *p* ≤ 0.01, *** = *p* ≤ 0.001.

## 3 Results

### 3.1 Manufacture and Characterization of MEW and Hybrid MEW‐FDM Constructs

**Figure 2:**
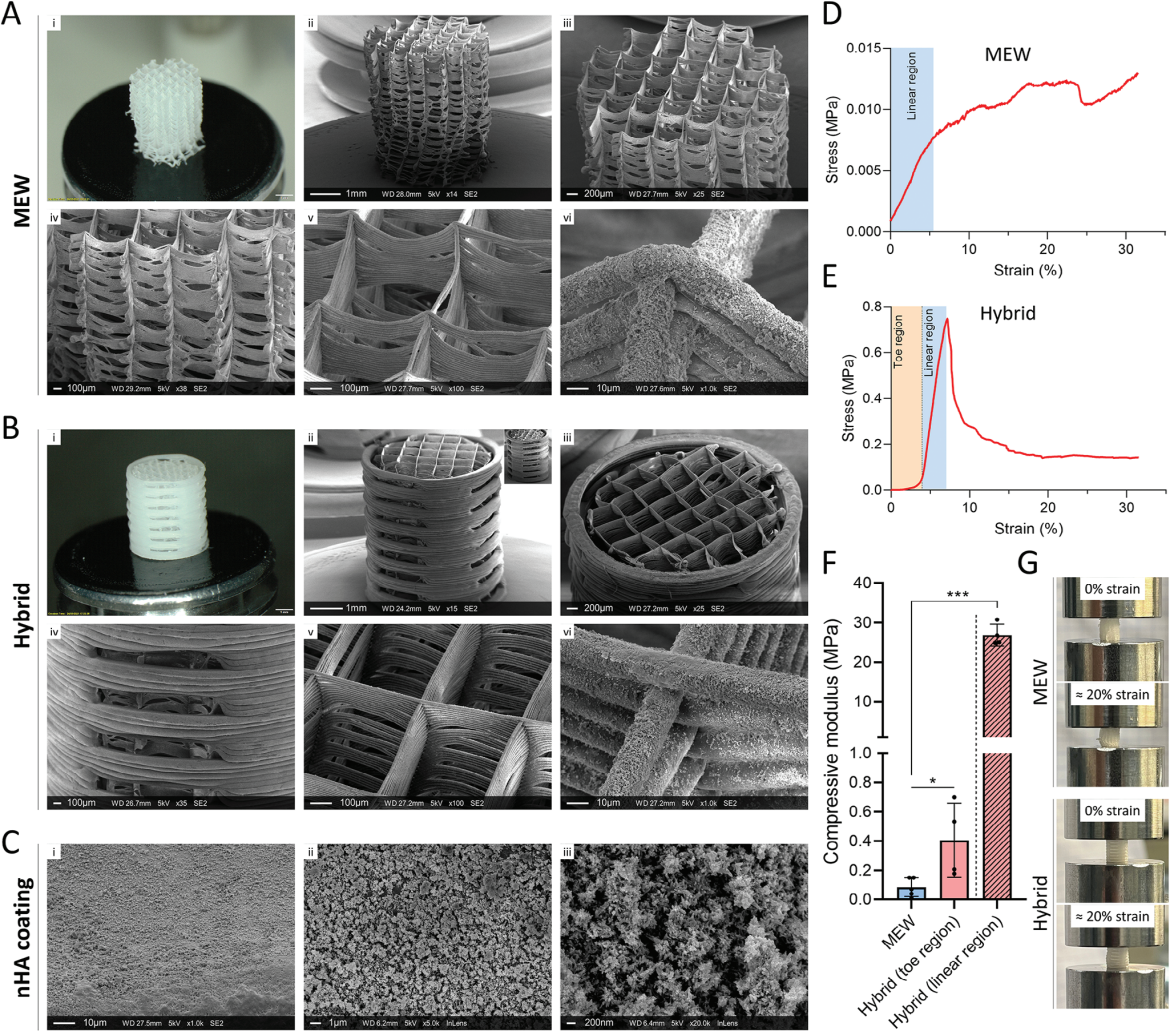
SEM imaging and characterization of scaffold groups. A‐i) Stereomicroscope and ii–vi) SEM imaging of MEW scaffold with fiber diameter of 18 µm. B‐i) Stereomicroscope and ii–vi) SEM imaging of hybrid scaffold with MEW fiber diameter of 18 µm and FDM housing fiber diameter of 200 µm. MEW fibers can be seen to slightly protrude through the side pores of the FDM shell, latching the assembly securely together. C) All scaffolds were coated with nnHA needles, as can be seen via high magnification SEM imaging. D) Stress–strain graph of representative MEW scaffold. E) Stress–strain graph of representative hybrid scaffold, showing the presence of a toe region and linear region. F) Calculation of compressive modulus of the initial linear region of MEW scaffolds, and of the toe region and following linear region in hybrid scaffolds. G) Images of MEW and hybrid scaffolds at 0% strain and ≈20% strain. Data presented as mean ± SD, *n* = 4–5, *p*‐values are calculated using unpaired T‐tests. *p ≤ 0.05, **p ≤ 0.01, ***p ≤ 0.001.

MEW scaffolds were successfully fabricated to a height of 4.75 mm. A stereomicroscope image of a MEW scaffold laser cut to a diameter of 3.75 mm is shown in **Figure** **2**A‐i, with an SEM image shown in Figure 2A‐ii. Accurate placement of fibers was maintained up to the scaffold height of 4.75 mm due to controlled incremental regulation of high voltage at each layer throughout the print, as shown in Figure 2A‐iii. A side view of the scaffold shows good stacking of fibers even toward the top of the scaffold (Figure 2A‐iv). Complete coverage of fibers with nnHA was achieved with 12 coatings, as can be seen in Figure 2A‐v,vi. Fibers have a diameter of 18 µm and scaffolds have a pore size of 600 µm. Hybrid scaffolds were also imaged in the same way (Figure 2B). A full view of the FDM shell can be seen in Figure 2Bi,ii, showing the relatively large pores which are located circumferentially around the long axis of the construct at 0°, 90°, 180°, and 270°. The diameter of FDM fibers is approximately 200 µm. The main image in Figure 2B‐ii shows the bottom of an MEW scaffold mounted in the FDM shell, while the inset shows the top view of an MEW scaffold in the FDM shell. It can be seen in Figure 2B‐iv how MEW fibers protrude slightly out of the side pores of the FDM shell, with this being the primary method of constraining the MEW scaffold and resulting in a highly secure assembly. The nnHA coating can also be seen on the FDM shell, in addition to on the MEW scaffold in Figure 2Bv,vi. Note that nnHA coating was conducted after assembly of the MEW scaffold and FDM shell, and this mineral coating may thus also help fuse the two components together. Further SEM imaging of the mineral coating shows the high density of nnHA (Figure 2Ci,ii), in addition to the needle morphology of the coating at the nano‐scale (Figure 2C‐iii). It should be noted that the hybrid scaffolds were much easier to handle compared to the MEW scaffolds, with the former being preferred by the surgeon during implantation in vivo.

Compressive mechanical testing revealed that the MEW scaffolds displayed an extremely low stiffness and a highly elastic behavior. Compression testing of MEW scaffolds demonstrated the presence of an initial linear region under low stresses which began to plateau at higher strains (Figure 2D). In contrast, hybrid scaffolds were characterized by an initial toe region followed by a much stiffer linear behavior until failure of the FDM shell (Figure 2E). The compressive modulus of the hybrid scaffolds (taken from the linear region of the stress‐strain curve) was over 300 times higher than the MEW scaffolds (Figure 2F). Images of scaffolds prior to compression, and at ≈20% strain, are shown in Figure 2G.

An in vitro growth factor release study was conducted on the nnHA coated hybrid+BMP2 scaffolds, where BMP2 was found to slowly release from the nnHA coating, with a total release of 8 µg (80%) after 21 days (Figure S3A,B, Supporting Information). After the release study, the same scaffolds were also stained for BMP2 using immunohistochemical staining and compared to control scaffolds (Figure S3C, Supporting Information). This confirmed that some BMP2 still remained on scaffolds after the release study, further demonstrating its capacity to effectively bind and slowly release this growth factor.

### 3.2 Hybrid Scaffolds Significantly Enhance Bone Healing

**Figure 3:**
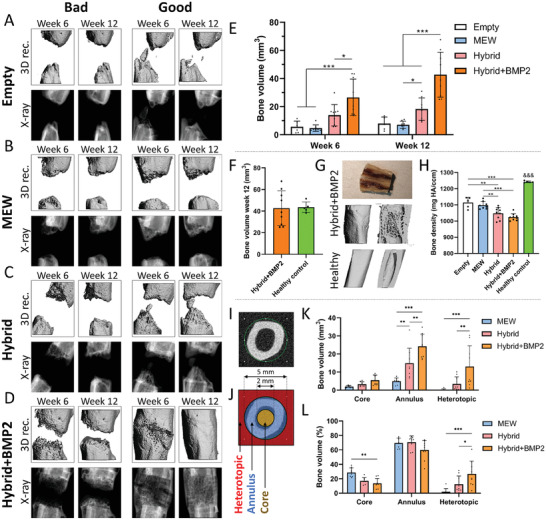
Analysis of bone healing using µCT imaging. Examples of good and bad healing are provided at 6 and 12 weeks for A) empty, B) MEW, C) hybrid, and D) hybrid+BMP2 groups. E) Bone volume within the 5 mm defect at week 6 and 12 showing enhanced healing, in particular with the hybrid and hybrid+BMP2 groups. F) Bone volume within the hybrid+BMP2 group at week 12 compared to healthy leg controls. G) Cross section of bone, µCT scan and cross section of µCT scan in the hybrid+BMP2 group at 12 weeks compared to a µCT scan and µCT scan cross section of a healthy control. H) Bone density at week 12 compared to healthy leg controls. I) µCT scan at healthy femur midsection, which is approximately 5 mm in diameter. J) Definition of heterotopic, annulus, and core regions based on scans of healthy rats. K) Total bone volume and L) percentage bone volume within the core, annulus, and heterotopic regions. Data presented as mean ± SD, *n* = 5–6 (empty group), *n* = 8–9 (MEW, hybrid, hybrid+BMP2 groups), *n* = 5 (Healthy control group), *p*‐values are calculated using two‐way ANOVA with Tukey's multiple comparisons post‐test for bone volume analyses, and one‐way ANOVA with Tukey's multiple comparisons post‐test for bone density analysis at week 12. *p ≤ 0.05, **p ≤ 0.01, ***p ≤ 0.001.

Healing of the femoral bone defects was first analyzed via µCT imaging. At 6 and 12 weeks, scans were taken for all rats, and representative 3D reconstructions and X‐ray images of rats with relatively lower levels of healing (“Bad”) and higher levels of healing (“Good”) are shown in **Figure** **3**. These classifications were based on bone volume and structure/organization of new bone. For the empty group, bone formed in a pointed and irregular manner, with large differences between rats and little evidence of bone regeneration (Figure 3A). In the MEW group, healing was more organized, with a flatter bone front and new bone growth being more organized and filling the volume of the scaffold (Figure 3B). Visible differences in bone healing were apparent between the hybrid scaffold and the MEW only scaffold, particularly at week 12 (Figure 3C). Complete bridging was nearly achieved with the hybrid implants, and more bone can visibly be seen compared to the MEW only group. The hybrid+BMP2 scaffolds supported the highest levels of bone regeneration, even in animals defined as “bad” healing where substantial new bone formation is observed by week 6, with bridging nearly occurring by week 12 (Figure 3D). At week 12, 6 out of the 9 rats displayed obvious and substantial defect bridging in this group, with the other 3 being very close. In several of the “good” healers in this group, complete bridging was seen at week 6 (Figure 3D). In the X‐ray view there appears to be a lower density of bone in the mid defect region at week 6. While substantial improvements were seen at week 12, the density of bone in the X‐ray view still appears to be relatively low, however subsequent analysis of the µCT data (see below) demonstrated the formation of well‐defined cortical bone. In all animals receiving the hybrid+BMP2 implant, new bone growth was generally constrained to the defect region, and no irregular or ectopic pieces of bone were present outside of the defect region in the surrounding tissue. Bone volume was quantified at week 6 and 12, where a trend of increasing bone volume from MEW to hybrid to hybrid+BMP2 was already apparent at week 6 (Figure 3E). Further changes were seen at week 12, with significant differences observed between the hybrid and MEW groups (2.6‐fold increase in hybrid vs MEW), and significantly larger bone volumes in the hybrid+BMP2 group compared to all other groups (2.3‐fold increase in hybrid+BMP2 vs hybrid). At week 12, an almost identical bone volume was observed in the hybrid+BMP2 group and healthy control femurs (Figure 3F). In Figure 3G, a photograph of a cross section of one hybrid+BMP2 rat is shown, in addition to a 3D reconstruction and cross section for a hybrid+BMP2 and healthy femur. Here, it was apparent that the diameter of the hybrid+BMP2 femur was larger, but that well defined cortical bone which was largely concentrated at the outer edges and integrated around the FDM shell fibers, in addition to a medullary cavity, had formed, with a structure similar to healthy bone. Bone density was also quantified (Figure 3H), where an inverse trend was present compared to bone volume, with the lowest density seen in the hybrid+BMP2 implants, likely due to the much greater amount of new bone present in the mid defect area of this group, which we would expect to be of lower density to normal bone until fully healed.

An analysis of the spatial distribution of new bone formation in the different scaffold groups was also conducted, with a 2 mm diameter region defining the core of the bone, a ring of outer diameter 5 mm and inner diameter 2 mm defining the annulus of the bone, with all regions outside of this 5 mm ring being defined as heterotopic (Figure 3I,J). In terms of total bone volume, significantly more bone was seen in the annulus and heterotopic regions of hybrid and hybrid+BMP2 scaffolds (Figure 3K). The hybrid scaffold supported significantly higher levels of annular bone formation compared to the MEW scaffold, demonstrating a functional role for the scaffold shell in enhancing bone healing. In the heterotopic region, there was more bone in the hybrid group (12%), and significantly more bone in the hybrid+BMP2 femurs (26%) compared to other groups. Importantly, bone in this region does not appear disorganized or “ectopic,” and femurs simply have a larger overall diameter in the region of healing, likely due to the presence of the FDM shell. The larger diameters appear to be due to the formation of a bone callus, which would likely remodel to a smaller diameter given further time.

### 3.3 Hybrid Scaffolds Provide Mechanical Support to the Defect site and Guide Bone Healing through the MEW Core

**Figure 4:**
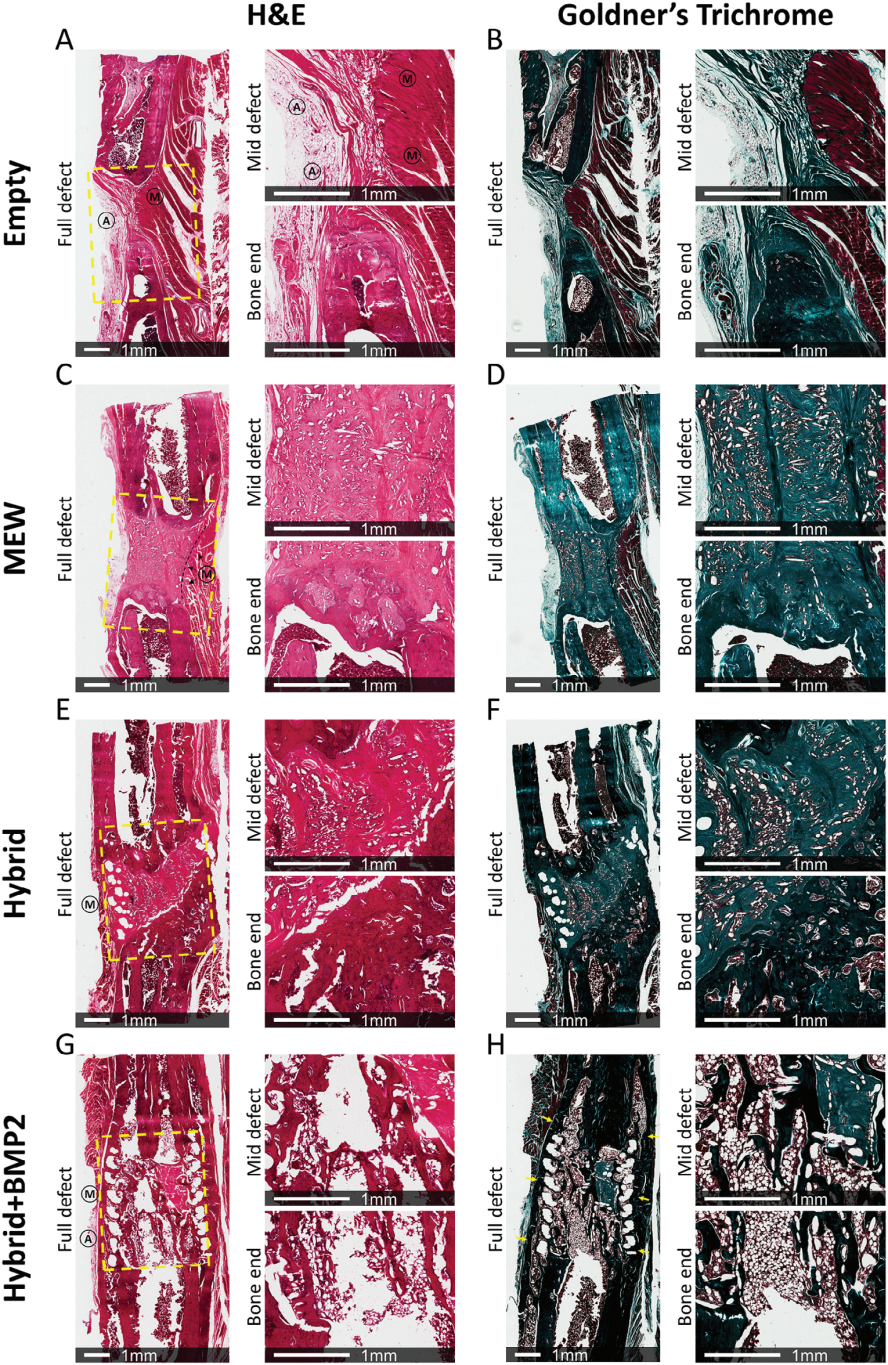
Histological staining of rat segmental defect study samples with good healing (relative to each group). A) H&E staining of the empty group. B) Goldner's trichrome staining of the empty group. C) H&E staining of the MEW group. Collapse of soft tissue into the defect is highlighted via a dashed line. D) Goldner's trichrome staining of the MEW group. E) H&E staining of the hybrid group. F) Goldner's trichrome staining of the hybrid group. G) H&E staining of the hybrid+BMP2 group. H) Goldner's trichrome staining of the hybrid+BMP2 group, where yellow arrows indicate the presence of calcified bone all along the edges of the defect. Dashed yellow lines indicate the defect region. The circled “M” indicates the presence of muscle tissue, while the circled “A” indicates the presence of adipose tissue. In the Goldner's trichrome stain, collagen is stained light green, mineralized collagen/calcified bone is stained in dark green, muscle is identified as striated tissue with a red stain, and blood vessels/red blood cells are stained in red.

Bone healing was further assessed by histological staining at 12 weeks, and as presented previously, examples of animals that displayed “good” (**Figure** **4**) and “bad” healing are provided (Figure S4, Supporting Information). In the empty group, the defect site appeared narrow, with irregular and pointed new bone growing into the defect (Figure 4A,B). There was a large quantity of muscle (indicated with “M”) and adipose (indicated with “A”) tissue present in the mid‐defect region. In the Goldner's trichrome stain, calcified bone is stained dark green, muscle is indicated by the striated red tissue, and blood vessels/red blood cells are stained in red. The morphology of healing was greatly improved with the MEW scaffold, with a more organized tissue in the defect (Figure 4C,D). In the MEW group, however, muscle tissue was still present within the defect site to a certain extent, with the soft MEW scaffold being compressed at the sides (Figure 4C). This presence of muscle and fat tissue in the empty and MEW groups reduced the space for new bone formation and is believed to impair regeneration. In the hybrid group, muscle tissue was constrained to the outside of the FDM shell and did not collapse through the large side pores of the shell (Figure 4E,F). New bone growth appeared to be directed by the internal MEW fibers, as seen by the dark green staining in Figure 4F. There was also some evidence of collagen tissue beginning to mineralize around the larger FDM fibers of the shell. As with the hybrid scaffold, muscle and adipose tissue was prevented from impinging into the defect site in the hybrid+BMP2 group (Figure 4G,H). Most notably, however, was the highly mineralized tissue distributed at the edges of the defect around the FDM shell (indicated by yellow arrows in Figure 4H). An advanced medullary cavity also appeared to have formed, as shown by lower quantity of mineralized bone in the middle of the defect, and a large amount of blood cell/marrow staining throughout the defect and into what was originally the bone ends after surgery. In some of the “bad” healers in the hybrid+BMP2 group (Figure S4G,H, Supporting Information), this cavity did not appear and new bone was primarily in the middle of the defect, indicating an earlier stage of healing. Also of interest was the similarity of healing morphology between the worst healers in the hybrid+BMP2 group and the best healers in the hybrid group. Safranin O staining was also carried out to detect cartilaginous tissue and possible healing via endochondral ossification, however, no positive staining was observed (Figure S5, Supporting Information).

### 3.4 The Relationship between Vascularization and Bone Healing Following Scaffold Implantation

**Figure 5:**
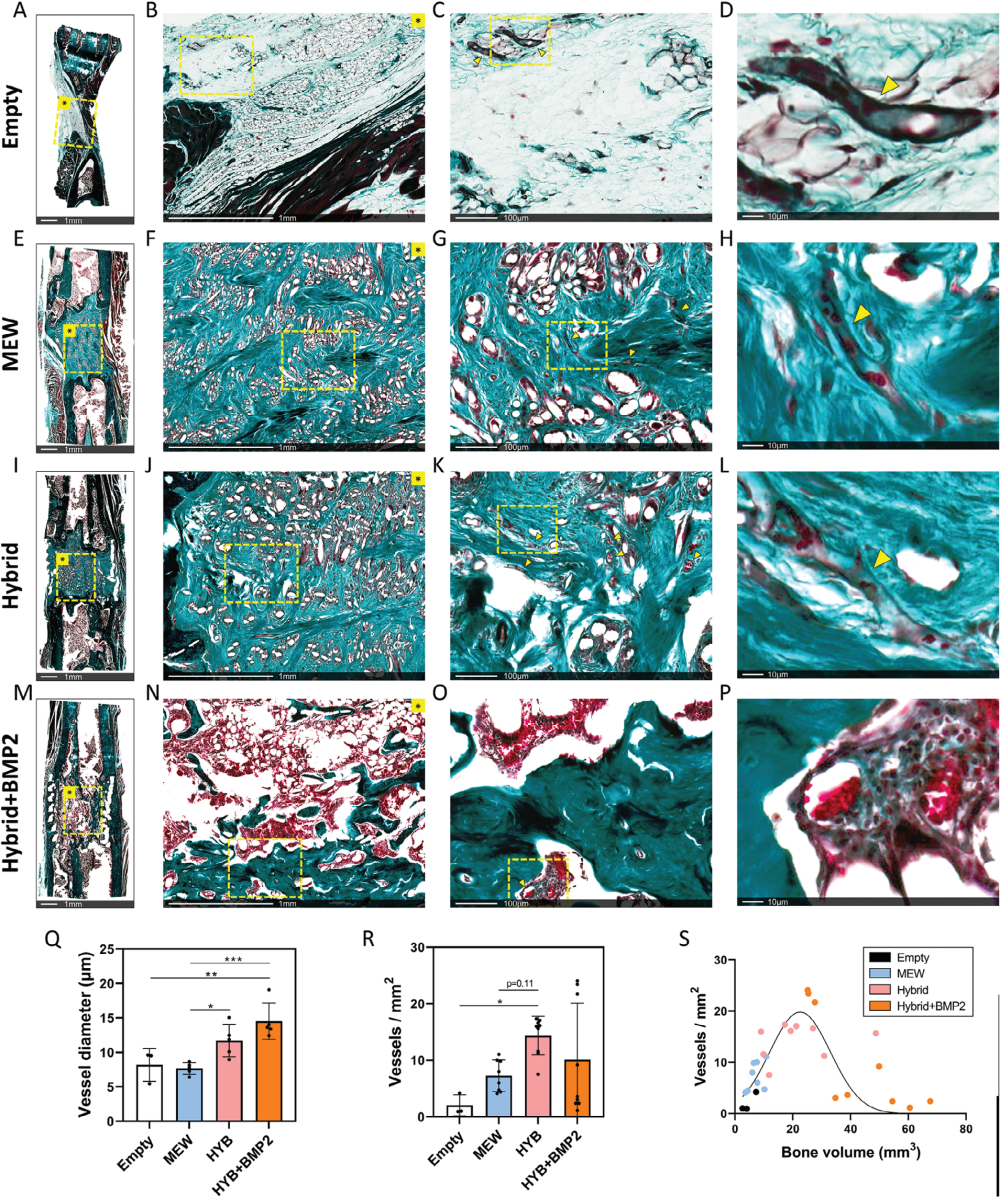
Analysis of vessel formation. A–D) Goldner's Trichrome staining of empty scaffold group. E–H) Goldner's Trichrome staining of MEW scaffold group. I–L) Goldner's Trichrome staining of hybrid scaffold group. M–P) Goldner's Trichrome staining of hybrid+BMP2 scaffold group. Q) Quantification of vessel diameter, showing increased vessel size in the hybrid and hybrid+BMP2 groups. R) Analysis of vessel density, showing a trend of increasing vessel number from empty–MEW–hybrid scaffold groups. The hybrid+BMP2 scaffold group had a large variation in vessel quantity between animals. S) Comparison between bone volume and vessel density for all groups. Here, the reason for the large variation in vessel quantity for the hybrid+BMP2 group can be seen, where enhanced healing is associated with lower number of vessels and the advanced formation of a medullary cavity filled with marrow. Examples of some vessels are indicated using yellow arrowheads. Dashed yellow boxes show region of focus for next element of figure in each group. Data is presented as mean ± SD. For vessel diameter, up to 20 random vessels were measured with biological replicates of *n* = 3 (empty group) and *n* = 5 (MEW, hybrid, hybrid+BMP2 groups). For vessel density, *n* = 3 (empty group) and *n* = 8‐9 (MEW, hybrid, hybrid+BMP2 groups). *p*‐values are calculated using one‐way ANOVA with Tukey's multiple comparisons post‐test. *p ≤ 0.05, **p ≤ 0.01, ***p ≤ 0.001.

Vessel formation was investigated at 12 weeks in all groups in a defined 2.5×2.0 mm region in the middle of defects, with vessel diameter and vessel number being quantified. Vessels were identified by their characteristic shape and the light red staining of red blood cells via Goldner's trichrome staining in the empty (**Figure** **5**A–D), MEW (Figure 5E–H), hybrid (Figure 5I–L), and hybrid+BMP2 (Figure 5M–P) groups. Of note is the very low vessel number in the empty group which is apparent visually in images even before quantification (Figure 5B,C). Also of note is the greatly different morphology of vessels in the hybrid+BMP2 group scaffolds, and in the better healers, the presence of a more continuous marrow cavity populated with red blood cells (Figure 5N). Vessel diameter was first quantified, with an average diameter of 8 µm in the empty and MEW groups. Interestingly, larger vessel diameters were seen in the hybrid and hybrid+BMP2 groups (Figure 5Q), with average values of 12 µm and 15 µm respectively. Vessel quantity in terms of vessels mm^−2^ was also quantified (Figure 5R), with an average of 2 vessels mm^−2^ in empty and 7 vessels mm^−2^ in MEW. Interestingly, the hybrid group had the largest number of vessels (14 vessels mm^−2^), with lower numbers in the hybrid+BMP2 group (10 vessels mm^−2^). Of note here is the very large standard deviation and spread of results (animal variability) in the hybrid+BMP2 group. It was found that some animals displayed the formation of a more advanced marrow cavity with mostly red blood cells being seen without defined vascular structures. In contrast, it was found that animals at an earlier stage of healing had more defined vessels. A non‐linear relationship was observed between vessel number (vessels mm^−2^) and bone healing (via bone volume in mm^3^) (Figure 5S). Bone volume and vessel number was positively correlated up to ≈25 mm^3^ of new bone, after which greater bone volumes displayed lower vessel numbers. Our observation here is that as healing becomes more advanced, a characteristic of lower vessel numbers in exchange for a more defined marrow cavity occurs.

## 4 Discussion

AM approaches are attractive for the fabrication of TE scaffolds due to their ability to form complex, pre‐defined architectures capable of eliciting specific biological responses in vitro and in vivo. While effective TE scaffolds can be fabricated with a single AM approach, there is an increasing interest in the use of hybrid strategies, which are attractive due to their ability to leverage the advantages of several AM approaches and form complex scaffolds to further enhance their functionality and ability to regenerate damaged tissue. In this study, we utilized FDM and MEW to form hybrid scaffolds for the regeneration of large bone defects. FDM was used to fabricate the outer shell of the scaffold, with the large and stiff fibers providing mechanical support to the defect site. MEW was used to fabricate the core of the scaffold, containing micro‐fibers supportive of osteogenesis and capable of guiding the bone healing process. Hybrid scaffolds were coated in nnHA, with one study group also being loaded with BMP2 via binding to the HA to further enhance bone healing. In a rat femoral defect model, we demonstrated that hybrid scaffolds enhanced bone formation compared to MEW only scaffolds. In the hybrid scaffold, the FDM shell provided additional structural support and guidance for bone healing, and prevented the encroachment of muscle and soft tissue into the defect site. In contrast, in the MEW only scaffold group, muscle and soft tissue caved into the defect site and additionally compressed the relatively soft scaffold structure, negatively affecting new bone formation. The hybrid scaffold loaded with BMP2 accelerated bone healing and enabled bridging of the defect in the majority of animals. These results demonstrate how a hybrid AM approach can be used to fabricate hierarchical scaffolds for bone TE applications.

The components of the hybrid scaffolds developed in this study were manufactured separately using FDM and MEW and assembled prior to mineral coating. While it is possible to print hybrid scaffolds in a single manufacturing process, this is not a trivial process, with an attractive alternative consisting of the use of different technologies which are subsequently combined to make hybrid constructs.^[^ ^15^, ^38^ ^]^ To simplify the process, we decided to manufacture the FDM shell and MEW core separately, and designed these components in such a way that they locked securely together when assembled. The shell was designed to mechanically support both the defect site and MEW scaffold within, while minimally influencing the ability of the MEW scaffold to guide bone healing. The shell also contained large pores around its periphery to maintain interaction between the inner defect site and its surroundings. The core–shell concept and the geometry of the MEW scaffold was developed based on the findings of a previous study,^[^ ^17^ ^]^ where we found that MEW scaffolds enhanced healing in segmental rat femoral defect studies. This geometric design was also guided by previous work with MEW scaffolds which showed that similarly sized open square pores of 500 µm facilitated good outcomes in terms of in vivo calvarial bone healing.^[^ ^24^ ^]^ There are some key design differences between the MEW scaffolds used in our previous work compared to the present study that may contribute to different regenerative outcomes. In the previous study, MEW scaffolds had a diameter and height of 4 mm and 4.8 mm respectively, and were designed as a standalone scaffold to fully fill the defect volume. In the present study, MEW scaffolds have a diameter and height of 3.75 mm and 4.75 mm, respectively, and were designed with a hybrid structure in mind, with the outer regions being comprised of an FDM shell. In the case of the control MEW only group used here, these smaller MEW scaffolds were implanted alone. This results in 13% less scaffold volume for bone healing in the MEW scaffolds here compared to our previous study,^[^ ^17^ ^]^ with the lost volume being in key places at the bone ends and annulus region due to the reduced scaffold height and diameter. However, despite the favorable healing outcomes of standalone MEW scaffolds in our previous study,^[^ ^17^ ^]^ the scaffolds were limited by their low stiffness. This made them difficult to handle and implant in defects and also resulted in scaffolds deforming due to the collapse of soft tissue into the defect sites. This may be less of an issue depending on the specific application of the scaffold, for example, in calvarial defects, where MEW scaffolds have previously shown promise for bone healing.^[^ ^24^ ^]^ Scaffolds manufactured by hybrid AM approaches may offer further benefits for applications where more mechanical support and protection is needed, for example, to prevent the collapse of soft tissue into the defect site or to provide protection and support against external impacts. Mechanical properties indeed have great importance for bone healing,^[^ ^27^, ^39^ ^]^ however there are many confounding factors,^[^ ^40^, ^41^ ^]^ with studies demonstrating favorable outcomes in terms of bone healing for both stiffer^[^ ^42^ ^]^ and softer^[^ ^43^ ^]^ scaffolds. Porosity in particular is an important factor which is interdependent with strength, with high porosity in particular being shown to be important for osteogenesis and bone healing.^[^ ^44^ ^]^ In a previous in silico analysis of bone healing, softer scaffolds with a stiffness of 0.1 and 0.4 MPa were predicted to yield higher bone volumes, which agreed with the findings of an in vivo study where softer scaffolds promoted superior new bone formation and enhanced angiogenesis compared to stiffer designs.^[^ ^43^ ^]^ Our hybrid scaffolds display non‐linear mechanical properties, being relatively soft in the toe‐region, which combined with the high porosity of the MEW core, may also be contributing to enhanced bone healing. We also propose that the relatively higher stiffness of the FDM shell compared to the MEW core may help promote the formation of well‐defined annular bone, as seen with the hybrid scaffold.

The hybrid scaffold supported significantly higher levels of new bone formation compared to the MEW only scaffold, with the majority of additional bone being located in the annular region of the defect. In the MEW group, soft tissue is seen histologically to collapse into the defect site. This soft tissue occupies a large proportion of the annulus region, compressing and altering the structure of the MEW scaffold and limiting space for bone growth. This is prevented by the stiffer outer shell of the hybrid scaffold. Previous studies have used a permeable collagen membrane around a scaffold to reduce fibrous tissue infiltration and enhance bone healing.^[^ ^45^ ^]^ In this previous study, the collagen membrane also had a direct influence on bone healing by acting as a scaffold for periosteal callus formation. In our study, the FDM shell may also have had a similar influence on bone healing, where we believe that in addition to the mechanical properties discussed previously, the additional presence of circumferential fibers in the annular region may provide guidance and support for the regeneration of new annular bone in addition to helping to initially focus healing of bone through the channel (including MEW core) of the shell. This is supported by both macroscopic images of excised bone (Figure 3G), in addition to histological findings (Figure 4H), which demonstrate the development of well‐defined annular bone formed around the fibers and integrated with the FDM shell. Further significant increases in bone volume after 12 weeks were seen with the hybrid+BMP2 scaffold group, with a fully bridged defect and well‐defined annular bone with a smooth outer surface and well developed medullary cavity. Excessive BMP2 concentrations and leakage outside of the implant site are known to contribute to adverse events such as disorganized and/or ectopic bone formation.^[^ ^33^, ^46^, ^47^ ^]^ In the present study, our strategy was to use CaP mineral as a means by which to bind and release BMP2 in a controlled manner. This has been shown to be a highly effective strategy for bone healing, with one study demonstrating that CaP coated collagen scaffolds supported a slower and more sustained release of BMP2 when compared to collagen only scaffolds without CaP, resulting in enhanced bone healing in vivo.^[^ ^48^ ^]^ Other similar approaches have also demonstrated the benefits of combining low doses of BMPs with CaP containing materials for bone regeneration.^[^ ^49^, ^50^ ^]^ Here, we loaded 10 µg of BMP2 per scaffold, which was bound to the mineral coating to enable a sustained release profile.^[^ ^25^ ^]^ The resulting healing outcomes were excellent, with little ectopic bone identified, verifying the efficacy of this CaP mediated delivery strategy. Bone density was found to be lowest in the hybrid+BMP2 group, however, it was still comparable to a previous study which utilized 3D printed BMP2 loaded scaffolds.^[^ ^51^ ^]^ We propose that the higher measured bone density in the empty and MEW group is due to the lower total volume of new bone present in these groups which can be sampled. This primarily includes bone nearer the bone defect ends, and a small portion of the bone defect ends themselves. Conversely, in the hybrid+BMP2 group there is a much larger amount of new bone present in the mid defect area, which we would expect to be of lower density until fully healed.

Defect vascularization was also found to depend on scaffold design, with both vessel diameter and quantity increasing following the implantation of hybrid scaffolds compared to MEW scaffolds. We propose that this is due to the larger effective pore size in hybrid scaffolds, which we believe remain as printed in vivo compared to the MEW group alone, where scaffolds are compressed in the defect, altering the pore architecture and size. We observed similar trends in our previous work, where the larger pores of an FDM scaffold (compared to MEW) supported higher levels of vascularization and the formation of larger diameter vessels,^[^ ^17^ ^]^ with another study also demonstrating that larger pores facilitated faster vessel ingrowth and greater levels of bone formation.^[^ ^52^ ^]^ A previous study also demonstrated that relatively low stiffness scaffolds of 0.67 MPa promoted higher levels of vascularization,^[^ ^43^ ^]^ a stiffness that is comparable to the toe‐region of the hybrid design. We also observed large animal‐to‐animal variability in vessel number within the hybrid+BMP2 group. To probe this further, we compared vessel number to bone volume, observing a non‐linear, bell‐shaped relationship, with the highest levels of vascularization seen when defects were approximately half‐way to bridging, and the lowest levels of vascularization seen in animals at both ends of the healing spectrum (i.e., the lowest and highest healing). Similar findings have been observed in mouse femoral defects, where vessel number increases in the weeks following surgery, before dropping again toward the end of the 10 week study.^[^ ^53^ ^]^ There is a known temporal relationship between bone formation and vascularization, termed angiogenic‐osteogenic coupling,^[^ ^54^, ^55^, ^56^ ^]^ whereby various factors including vascular endothelial growth factor (VEGF) are upregulated during fracture healing resulting in increased vessel formation.^[^ ^57^ ^]^ For animals with advanced healing in the hybrid+BMP2 group, a defined medullary cavity also developed, which was continuous from the native bone ends throughout the length of the scaffold. This cavity was filled with marrow and very few defined vessels could be identified. We believe that the soft, highly porous nature of the MEW core facilitates the early formation of an appropriate microvasculature for bone healing, before remodeling of this early bone into marrow at advanced stages of healing with an associated reduction in vasculature. Simultaneously, the stiff outer FDM shell may support this process by supporting the defect and MEW scaffold within, while also facilitating the formation of a well‐defined outer region of annular bone.

## 5 Conclusions

In conclusion, we have developed a new hybrid scaffold concept comprising a MEW core and FDM shell for the healing of large bone defects. We found that compared to MEW scaffolds alone, hybrid scaffolds enhanced defect vascularization and supported significantly higher levels of new bone formation. Furthermore, the hybrid+BMP2 scaffolds further accelerated bone healing and facilitated the full bridging of defects with a developed and open medullary cavity, well defined annular bone, and with little or no evidence of abnormal ectopic bone formation. These results highlight how hybrid AM approaches may be used to significantly enhance healing outcomes in large bone defects compared to the use of a single AM approach, and particularly demonstrate the potential of hybrid MEW‐FDM constructs for this application.

## Conflict of Interest

The authors declare that this research has been co‐funded by Johnson & Johnson 3D Printing Innovation and Customer Solutions, Johnson & Johnson Services Inc.

## Author Contributions

K.F.E.: Conceptualization and development of methodology, manuscript writing, MEW and FDM scaffold design, fabrication of scaffolds, SEM imaging, data analysis, involved in all aspects of animal study (animal procedures, µCT imaging and analysis, histology). P.P.: Involved in all aspects of animal study (animal procedures, µCT imaging and analysis, histology). R.B.: FDM scaffold design, involved in all aspects of animal study (animal procedures, µCT imaging and analysis, histology). F.C.: involved in BMP2 release study and immunohistochemistry. X.B.: Fabrication of scaffolds, involved in BMP2 release study and immunohistochemistry. B.T.: Mechanical testing of scaffolds. R.P.: Laser cutting of MEW scaffolds and optimizing settings for hybrid scaffold assembly. O.G.: Conceptualization and development of methodology, project supervision. D.J.K.: Conceptualization and development of methodology, project supervision, manuscript writing. All authors read and approved the final manuscript.

## Supporting information

Supporting Information

## Data Availability

The data that support the findings of this study are available from the corresponding author upon reasonable request.
