# Supplementary material for: Integrating Melt Electrowriting and Fused Deposition Modeling to Fabricate Hybrid Scaffolds Supportive of Accelerated Bone Regeneration
Source: Adv Healthc Mater. 2023 Nov 16;13(3):2302057. doi: 10.1002/adhm.202302057 (PMC11468945; doi:10.1002/adhm.202302057)
Supplement: Supplementary file 1 — Supporting Information [file ADHM-13-2302057-s001.pdf]

ADVANCED HEALTHCARE MATERIALS

Supporting Information

for *Adv. Healthcare Mater.*, DOI 10.1002/adhm.202302057

Integrating Melt Electrowriting and Fused Deposition Modeling to Fabricate Hybrid Scaffolds Supportive of Accelerated Bone Regeneration

*Kian F. Eichholz, Pierluca Pitacco, Ross Burdis, Farhad Chariyev-Prinz, Xavier Barceló, Brooke Tornifoglio, Ryan Paetzold, Orquidea Garcia and Daniel J Kelly**

Supplementary information

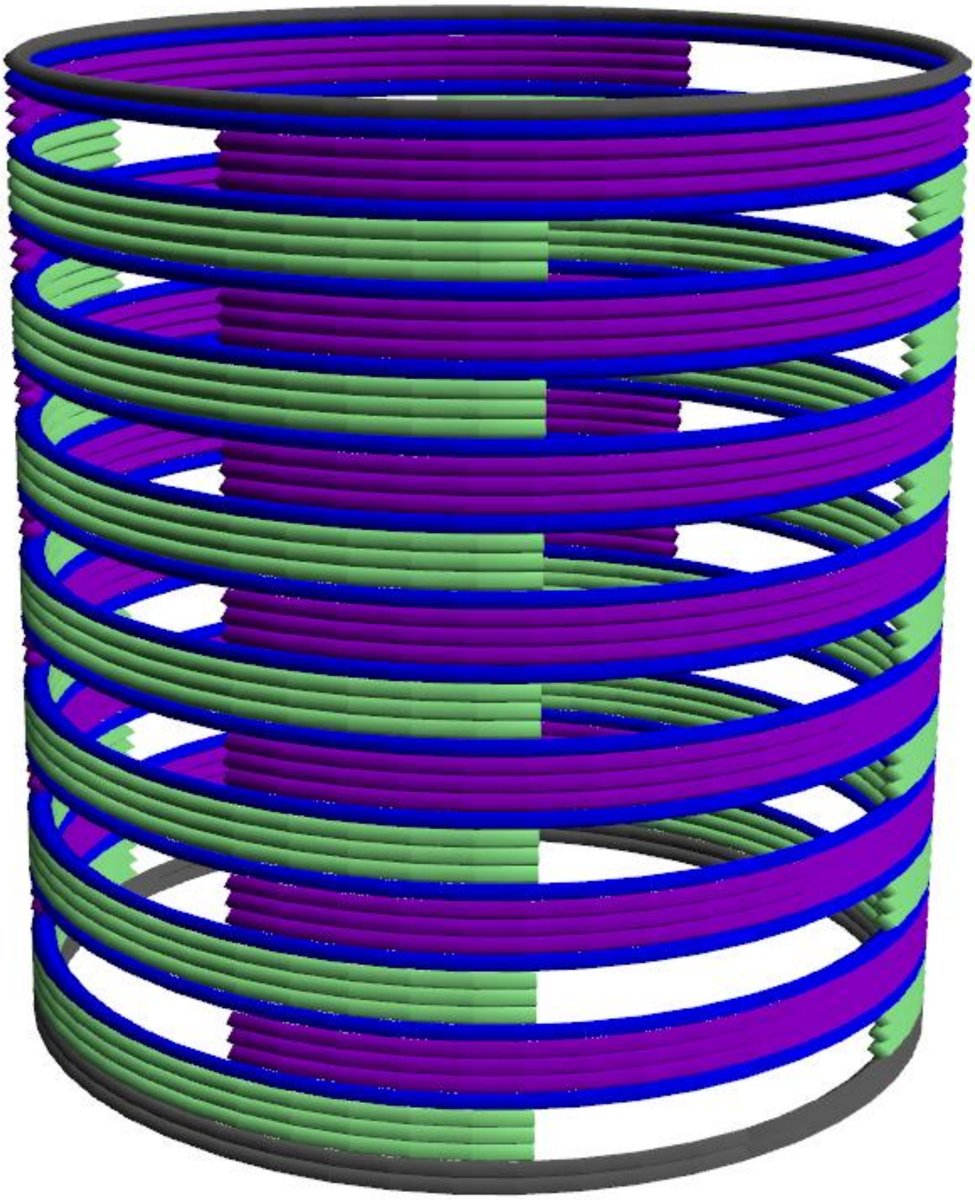

Figure S 1 Print path of FDM shell, showing placement of material.

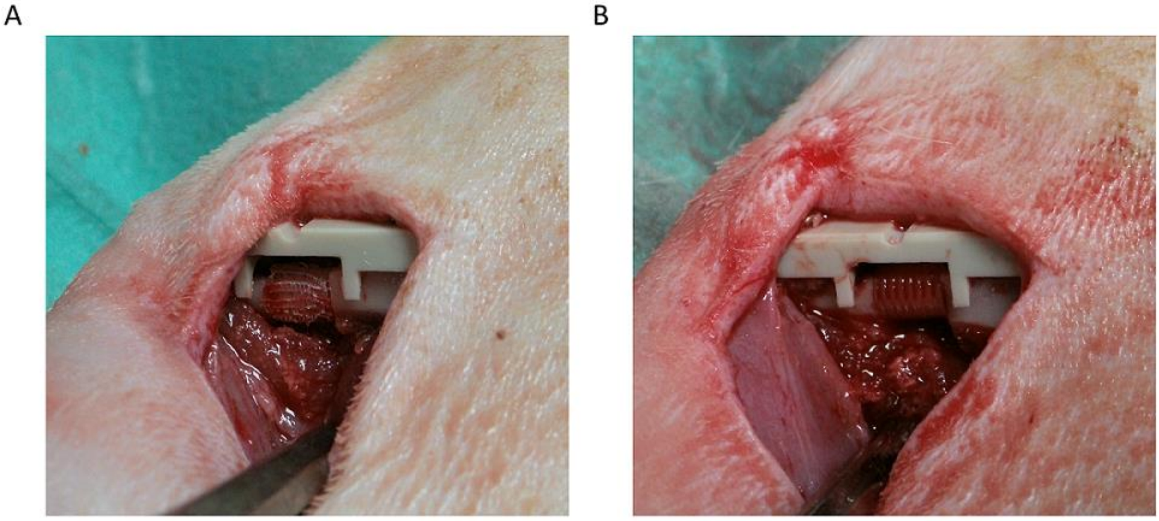

Figure S 2 Photos taken after implantation of scaffolds in 5 mm segmental femoral defects. A MEW scaffold. **B** Hybrid scaffold.

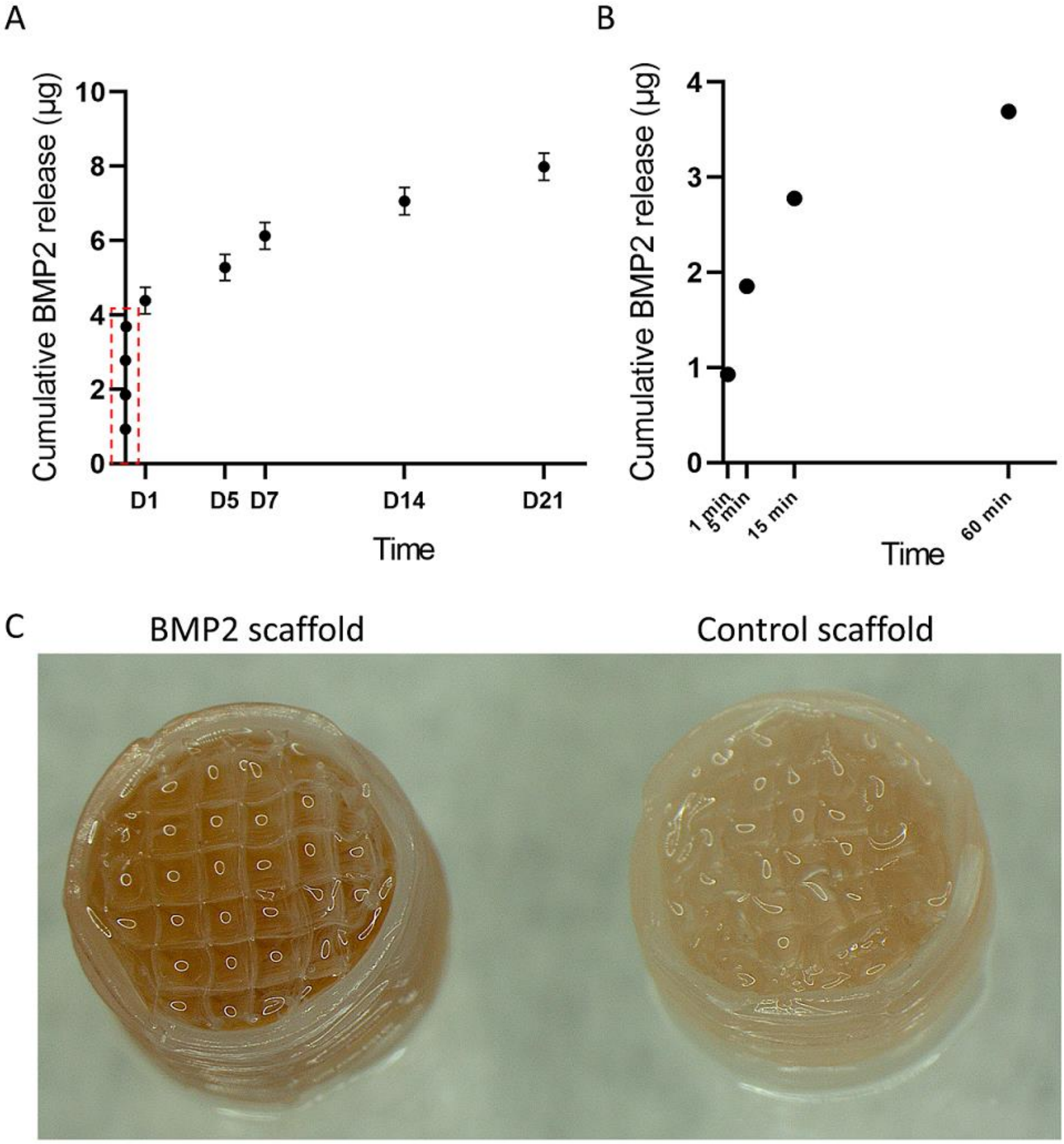

Figure S 3 A Cumulative quantity of BMP-2 released over time. **B** Early release profile, with a focus on the first four time-points as highlighted in **A**. **C** Immunohistochemical staining of BMP2 loaded scaffold after the release study, showing that BMP2 is still present after this study. A control scaffold with no BMP2 loading was also stained as a comparison. Data presented as mean ± SD, n=4.

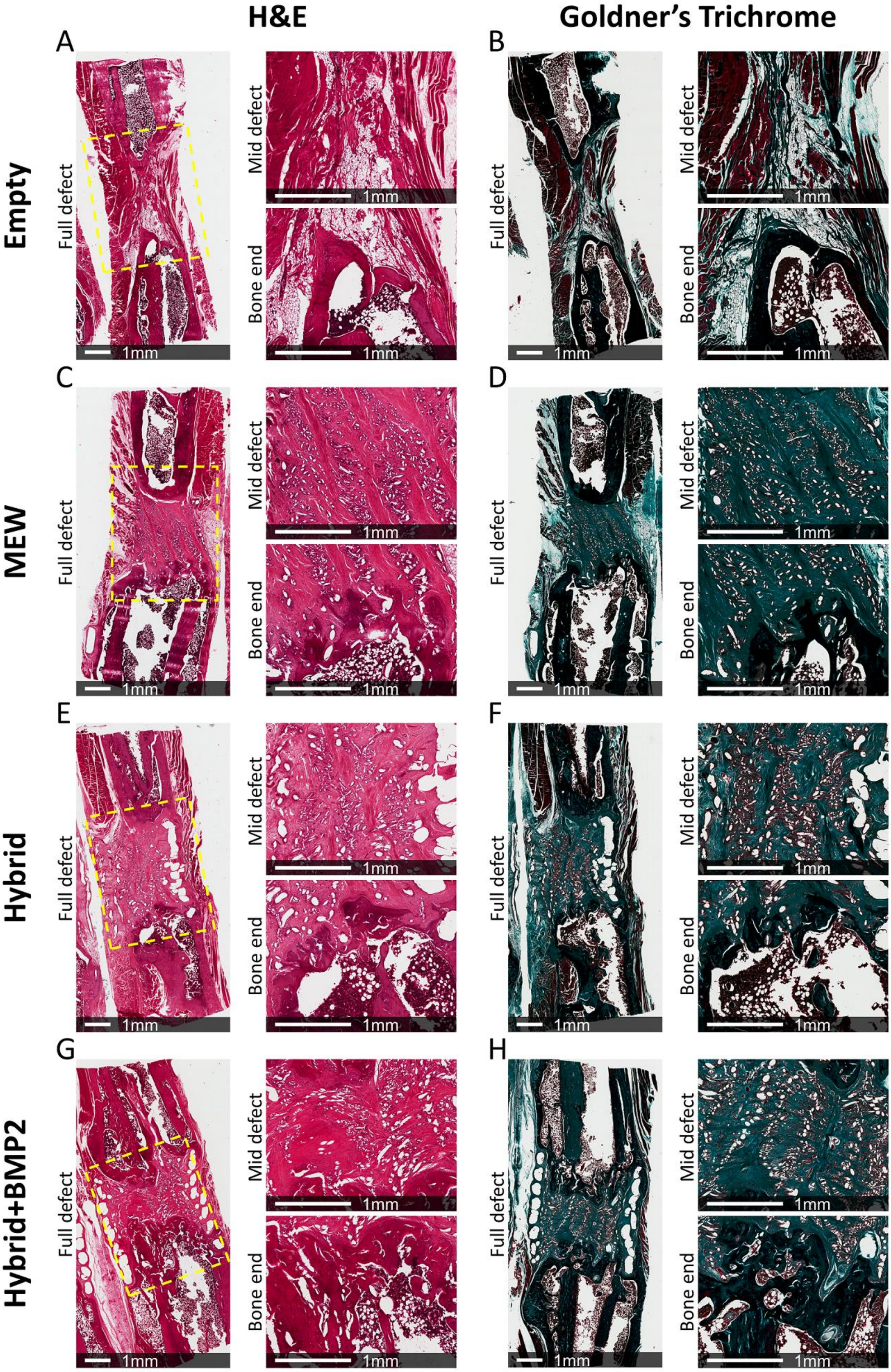

H&E
Goldner's Trichrome
A
B
C
D
E
F
G
H
Empty
MEW
Hybrid
Hybrid+BMP2
Full defect
Mid defect
Bone end
1mm

Figure S 4 Histological staining of rat segmental defect study samples with bad healing (relative to each group). A H&E staining of the empty group. **B** Goldner's trichrome staining of the empty group. **C** H&E staining of the MEW group. **D** Goldner's trichrome staining of the MEW group. **E** H&E staining of the Hybrid group. **F** Goldner's trichrome staining of the Hybrid group. **G** H&E staining of the Hybrid+BMP2 group. **H** Goldner's trichrome staining of the Hybrid+BMP2 group. Dashed yellow lines indicate the defect region.

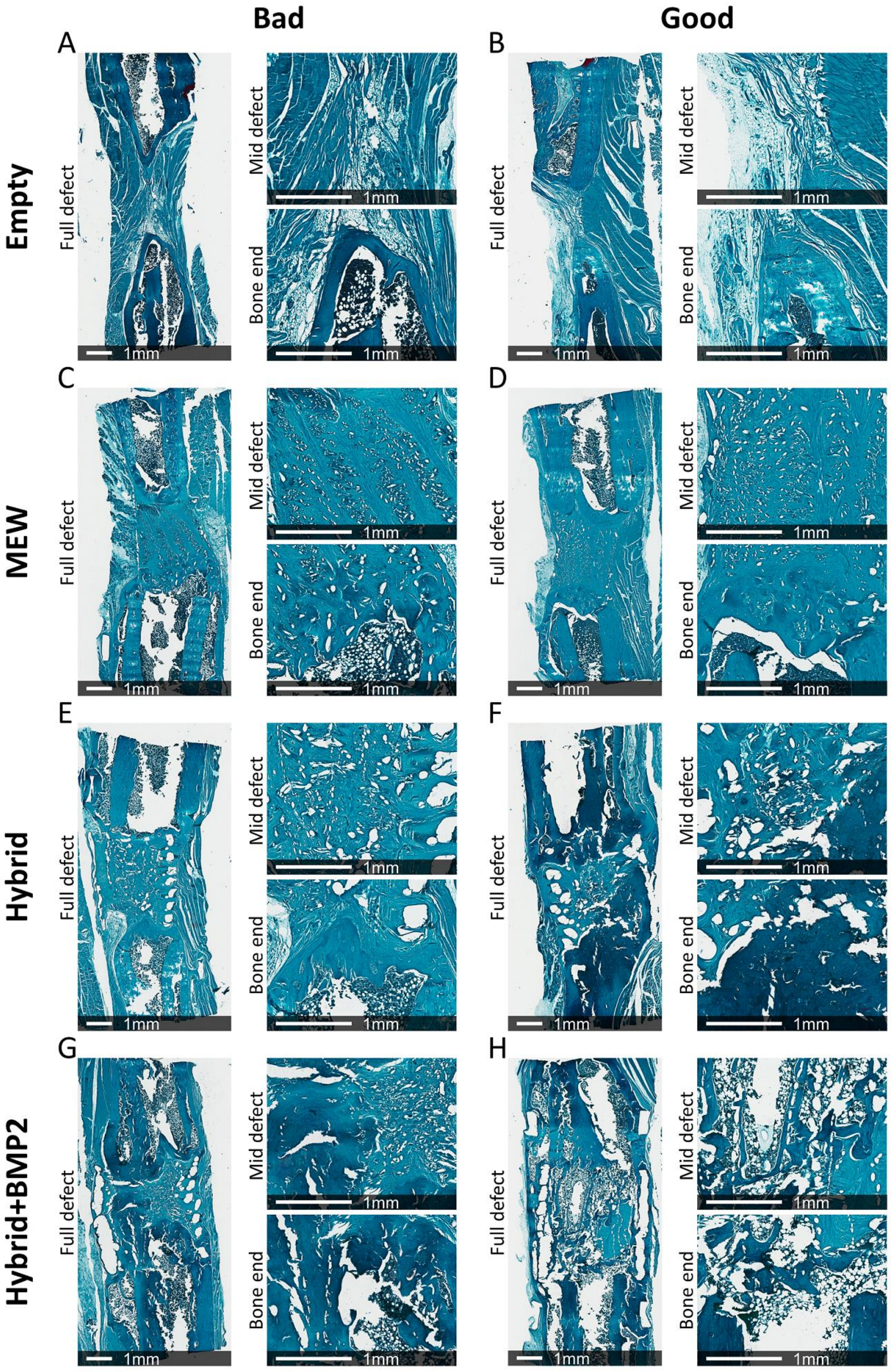

Bad
Good
A
B
C
D
E
F
G
H
Empty
MEW
Hybrid
Hybrid+BMP2
Full defect
Mid defect
Bone end
1mm

Figure S 5 Histological safranin O of rat segmental defect study samples. A Staining of empty group with bad healing. **B** Staining of empty group with good healing. **C** Staining of MEW group with bad healing. **D** Staining of MEW group with good healing. **E** Staining of Hybrid group with bad healing. **F** Staining of Hybrid group with good healing. **G** Staining of Hybrid+BMP2 group with bad healing. **H** Staining of Hybrid+BMP2 group with good healing. No appreciable safranin O staining was seen in any groups.
